# National and State Vaccination Coverage Among Adolescents Aged 13–17 Years — United States, 2012

**Published:** 2013-08-30

**Authors:** C. Robinette Curtis, David Yankey, Jenny Jeyarajah, Christina Dorell, Shannon Stokley, Jessica MacNeil, Susan Hariri

**Affiliations:** Immunization Services Div; Div of Bacterial Diseases, National Center for Immunization and Respiratory Diseases; Div of Sexually Transmitted Diseases, National Center for HIV/AIDS, Viral Hepatitis, STD, and TB Prevention, CDC

At ages 11 through 12 years, the Advisory Committee on Immunization Practices (ACIP) recommends that preteens receive 1 dose of tetanus, diphtheria, and acellular pertussis (Tdap) vaccine, 1 dose of meningococcal conjugate (MenACWY) vaccine,* and 3 doses of human papillomavirus (HPV) vaccine (1–3). ACIP recommends administration of all age-appropriate vaccines during a single visit (4). ACIP also recommends that pre-teens and older adolescents receive an annual influenza vaccine as well as any overdue vaccines (e.g., varicella) (1). To monitor vaccination coverage among persons aged 13–17 years,† CDC analyzed data from the National Immunization Survey–Teen (NIS-Teen). This report highlights findings of that analysis. From 2011 to 2012, coverage increased for ≥1 Tdap vaccine dose§ (from 78.2% to 84.6%), ≥1 MenACWY vaccine dose (from 70.5% to 74.0%) and, among males, ≥1 HPV vaccine dose (from 8.3% to 20.8%). Among females, vaccination coverage estimates for each HPV vaccine series dose were similar in 2012 compared with 2011. Coverage varied substantially among states. Regarding *Healthy People 2020* targets for adolescents (5), 36 states achieved targets for Tdap, 12 for MenACWY, and nine for varicella vaccine coverage. Large and increasing coverage differences between Tdap and other vaccines recommended for adolescents indicate that substantial missed opportunities remain for vaccinating teens, especially against HPV infection (6). Health-care providers should administer recommended HPV and meningococcal vaccinations to boys and girls during the same visits when Tdap vaccine is given. In addition, whether for health problems or well-checks, providers, parents, and adolescents should use every health-care visit as an opportunity to review adolescents’ immunization histories and ensure that every adolescent is fully vaccinated.

NIS-Teen identifies persons aged 13–17 years in the 50 states, the District of Columbia, selected areas,¶ and the U.S. Virgin Islands** using a random-digit–dialed sample of landline and, since 2011, cellular telephone numbers.†† Survey respondents are parents or guardians of teens aged 13–17 years who provide information about their children’s sociodemographic characteristics and vaccination providers. After receiving consent from respondents, questionnaires are mailed to all identified providers to obtain data from medical records, so that composite, provider-reported immunization histories can be analyzed.§§ In 2012, national estimates included 19,199 adolescents (9,058 females; 10,141 males).¶¶ Details regarding NIS-Teen methodology, including methods for synthesizing provider-reported immunization histories and weighting, have been described.*** T-tests were used to assess vaccination coverage differences by survey year, age, sex, race/ethnicity, and poverty status for all vaccines included in this report. Weighted linear regression was used to assess coverage trends for vaccines recommended routinely for adolescents since 2005–2006 (i.e., Tdap, MenACWY, and among females, HPV vaccine). Results were considered statistically significant at p<0.05.

## National Vaccination Coverage

Vaccination coverage trends differ substantially for the three vaccines routinely recommended for adolescents since 2005–2006 (Figure). During 2006–2012, coverage for ≥1 Tdap vaccine dose and ≥1 MenACWY vaccine dose increased steadily, with annual average increases of approximately 12.0 (95% confidence interval [CI] = 9.9–14.0) and 10.1 (CI = 7.5–12.6) percentage points, respectively. Since 2009, the national estimate for ≥1 MenACWY vaccine dose has been lower than the estimate for ≥1 Tdap vaccine dose, and the difference in coverage between the two vaccines is widening (Figure). From 2011 to 2012, while ≥1 Tdap vaccine dose coverage increased 6.4 percentage points, coverage for ≥1 MenACWY vaccine dose increased only 3.5 percentage points. During 2007–2011, coverage for ≥1 HPV vaccine dose among females lagged behind estimates for Tdap and MenACWY vaccines, increasing on average 6.1 (CI = 3.3–8.9) percentage points each year. However, in 2011 and 2012, HPV vaccination rates among females did not increase (Figure, Table 1). Overall, HPV vaccination series completion among females was lower in 2012 compared with 2011.††† Compared with 2011 coverage rates, 2012 coverage estimates among males for HPV vaccine doses were higher (Figure, Table 1), but ≥1 dose coverage was lower (p<0.05) in 2012, the first survey year following the routine recommendation for males (3), than that achieved for females by 2007 (Figure) (7), the first survey year following licensure of the quadrivalent HPV vaccine for administration to females (2).

Among vaccines recommended for adolescents if not previously administered, coverage remained >90% for ≥2 MMR vaccine doses and ≥3 hepatitis B vaccine doses. Varicella vaccination coverage increased significantly for ≥1 and ≥2 doses (Table 1).

What is already known on this topic?

At ages 11 through 12 years, the Advisory Committee on Immunization Practices (ACIP) recommends that preteens receive 1 dose of tetanus, diphtheria, and acellular pertussis (Tdap) vaccine, 1 dose of meningococcal conjugate (MenACWY) vaccine, and 3 doses of human papillomavirus (HPV) vaccine. ACIP recommends administration of all age-appropriate vaccine doses during a single visit. During 2006–2011, national coverage for ≥1 Tdap vaccine dose and ≥1 MenACWY vaccine dose increased steadily, with Tdap vaccine coverage in 2011 reaching national target levels for adolescents. During 2007–2011, coverage for ≥1 HPV vaccine dose among females lagged behind estimates for Tdap and MenACWY vaccination. In 2011, ACIP recommended routine HPV vaccination for males.

What is added by this report?

From 2011 to 2012, vaccination coverage among U.S. adolescents increased to 84.6% for ≥1 dose of Tdap vaccine, 74.0% for ≥1 dose of MenACWY vaccine, and, among males, to 20.8% for ≥1 dose of HPV vaccine. At 53.8%, vaccination coverage for ≥1 dose of HPV vaccine among females in 2012 was statistically unchanged from 2011, and only one third of female teens received all 3 recommended doses of the HPV series. Vaccination coverage levels continued to vary widely among states. Although the difference in vaccination coverage between Tdap and MenACWY has been increasing since 2009, national progress toward achievement of *Healthy People 2020* targets continues for Tdap and MenACWY vaccines.

What are the implications for public health practice?

Large and increasing coverage differences between Tdap and other vaccines recommended for adolescents show that many opportunities are being missed to vaccinate boys and girls, especially against HPV infection. Health-care providers should administer recommended HPV and meningococcal vaccinations to teens during the same visits when Tdap vaccine is given. Providers, parents, and adolescents also should use every health-care visit as an opportunity to review adolescents’ immunization histories and ensure that every adolescent is fully vaccinated.

## Vaccination Coverage by Selected Characteristics

In 2012, vaccination coverage rates were similar across age groups for Tdap, MenACWY, HPV (among males), MMR, and hepatitis B vaccines (Table 1). Older teens had lower varicella ≥1 and ≥2 dose coverage than younger age groups. Among females, HPV vaccination coverage increased by an average of approximately 4–6 percentage points per year of age for ≥1, ≥2, ≥3 doses and series completion (p<0.05); however, even among females aged 17 years (the most highly vaccinated age group), only 44.5% had received ≥3 doses.

In 2012, with the exception of HPV vaccination (Table 1), estimates were similar for both sexes for Tdap, MenACWY, MMR, hepatitis B, and varicella vaccination coverage measures. Tdap (≥1 dose) vaccination coverage was similar across poverty levels§§§ and racial/ethnic groups (Table 2). MenACWY (≥1 dose) vaccination coverage was similar across poverty levels; however, whites had lower coverage than other racial/ethnic groups. HPV vaccination coverage was higher for those living below poverty level for ≥1 and ≥2 doses among females and ≥1, ≥2, ≥3 doses among males; however, among females, series completion was higher among those living at or above poverty level. Compared with whites, HPV vaccination coverage rates for Hispanics were higher for ≥1 and ≥2 doses of vaccine among females and ≥1, ≥2, ≥3 doses among males. Among males, coverage for ≥1 and ≥2 HPV vaccine doses was higher for blacks compared with whites, but 3-dose series completion was lower. Among females, HPV vaccine series completion was lower for Hispanics and blacks compared with whites. Coverage for ≥2 doses MMR vaccine and ≥3 doses hepatitis B vaccine differed by poverty level and was lower for Hispanics compared with whites. Varicella vaccine coverage (≥2 doses) was lower for those living below the federal poverty level.

## State Vaccination Coverage

Coverage estimates for Tdap, MenACWY, and HPV vaccines varied widely among states. Coverage for ≥1 Tdap vaccine dose ranged from 53.5% (Mississippi) to 96.3% (New Hampshire), and for ≥1 MenACWY vaccine dose, from 37.5% (Arkansas) to 94.3% (Rhode Island) (Table 3). Among females, coverage for ≥1 HPV vaccine dose varied from 39.4% (Florida) to 73.7% (Rhode Island), and for ≥3 HPV vaccine doses, from 12.1% (Mississippi) to 57.7% (Rhode Island). Among males, coverage for ≥1 HPV vaccine dose ranged from 11.2% (Wyoming) to 55.2% (Rhode Island). Regionally, vaccination coverage was highest overall in the Northeast (Table 3). Among males, vaccination coverage estimates for each HPV vaccine series dose and HPV series completion were similar across regions.

## Healthy People 2020 Targets

The *Healthy People 2020* national targets for vaccination coverage among adolescents by ages 13–15 years are 80.0% for ≥1 Tdap dose, ≥1 MenACWY dose, and ≥3 HPV doses (among females), and 90.0% for ≥2 varicella doses (5). Among adolescents aged 13–15 years, vaccination coverage in 2012 was 85.3% (CI = 84.1–86.5) for ≥1 Tdap dose, 73.8% (CI = 72.3–75.2) for ≥1 MenACWY dose, 28.1% (CI = 26.1–30.2) for ≥3 HPV doses (among females), and 76.8% (CI = 75.1–78.4) for ≥2 varicella doses. Measures for Tdap, MenACWY, and varicella vaccines increased by 2.3–5.0 percentage points from 2011 to 2012; HPV vaccine (≥3 doses) coverage remained unchanged. Based on point estimates, 36 states met or exceeded national Tdap vaccination coverage targets, 12 met or exceeded MenACWY targets, and nine met or exceeded varicella targets. No state met the national target for HPV vaccination coverage among females.

### Editorial Note

National progress toward achievement of *Healthy People 2020* targets for adolescents has been observed for Tdap, MenACWY, and varicella vaccines; however, at only 28.1%, national coverage for ≥3 HPV vaccine doses among females aged 13–15 years remains far short of the *Healthy People 2020* target of 80%. In contrast, in 2012, coverage estimates among teens aged 13–15 years for ≥1 Tdap vaccine dose and ≥1 MenACWY vaccine dose were 85.3% and 73.8%, respectively, demonstrating that 80% vaccination coverage is achievable among adolescents. Among teens aged 13–17 years, the gap widened between Tdap and MenACWY vaccination coverage. Although age-related disparities were not observed in 2012 for many vaccines, age-related disparities were present for older adolescents for varicella and, among younger females, for HPV vaccination coverage (e.g., coverage for ≥3 HPV vaccine doses was more than 24 percentage points lower among females aged 13 years compared with those aged 17 years). Since reporting of HPV vaccination estimates among females began in 2007 with an initial ≥1 HPV vaccine dose coverage estimate of 25.1% (7), coverage rates for ≥1 HPV vaccine dose have increased only modestly compared with estimates for ≥1 Tdap vaccine dose and ≥1 MenACWY vaccine dose. However, from 2011 to 2012, HPV dose-specific vaccination rates among females did not increase at all, and series completion actually decreased. Following routine recommendations for males in 2011 (3) and females in 2006 (2), respectively, the initial coverage in 2012 for ≥1 HPV vaccine dose for males was lower than initial coverage for females (7). Differences in vaccination coverage underscore that clinicians and parents are missing opportunities to administer HPV, MenACWY, and varicella vaccinations during visits when Tdap vaccine is given.

Vaccination coverage estimates remained widely variable by state and vaccine. Differing state school vaccination requirements for Tdap, MenACWY, and varicella vaccines, respectively, might have fostered increased coverage for these vaccines (8). For entry into nonresidential middle schools during the 2012–13 school year, 40 states required Tdap vaccination.¶¶¶ Increased Tdap vaccination coverage also might have been influenced by provider and parent awareness that, in 2012, most states reported increased pertussis cases or outbreaks.****

As with other vaccines recommended for the civilian population of the United States, ACIP recommends Tdap, MenACWY, and HPV vaccines for the youngest age group at risk for the vaccine-preventable diseases for whom safety and efficacy of the particular vaccines have been shown (1,4). ACIP recommends administration of all age-appropriate vaccines during a single visit (4). For example, during a single visit, a healthy child aged 11 years should routinely receive recommended doses of Tdap, MenACWY, and HPV vaccines; then, before leaving the provider’s practice settings, two subsequent visits within 6 months should be scheduled for completion of the HPV vaccine series as recommended.

Other recommended strategies for increasing vaccination coverage, including HPV vaccination among females, have been well-described (6,8,9), but many have not been widely adopted. Clinicians should provide strong, clear, consistent vaccination recommendations to adolescents and their parents or guardians (6). Clinicians, public health agencies, and other stakeholders can also improve vaccination rates by reducing out-of-pocket vaccination costs for patients and their families (8). Through enrolled vaccination providers, the Vaccines for Children (VFC) program provides vaccines for uninsured, Medicaid-eligible, and other children through age 18 years whose families might not otherwise be able to afford vaccines.†††† HPV vaccination coverage was generally higher among teens living in poverty, which might reflect the VFC program’s effectiveness at reaching these young persons; however, series completion rates were lower among teens living in poverty, suggesting that other barriers need to be identified and addressed for this vulnerable population.

Implementation of the Patient Protection and Affordable Care Act of 2010§§§§ also offers opportunities to improve vaccination coverage among children and adolescents. Under the law, nongrandfathered private health plans must offer, at no cost to beneficiaries, vaccines that are recommended by ACIP. Similarly, qualified health plans on the new health exchanges that go into effect starting in 2014 must offer ACIP-recommended vaccines at no cost to beneficiaries.

The findings in this report are subject to at least three limitations. First, household response rates were 23.6% (cellular phone households) and 55.1% (landline households), respectively. Only 56.4% (cellular telephone) and 62% (landline) of completed household interviews also had adequate provider-verified vaccination data. After weighting adjustments, bias from nonresponse and exclusion of households without telephones might have remained. Coverage estimate increases of approximately 3 percentage points for Tdap, 2 for MenACWY, and 6 among females for HPV vaccination initiation might have resulted, based on a total survey error model including comparison to provider-reported data collected from a sample of National Health Interview Survey participants. Estimates of bias do not include errors in vaccination status (e.g., under ascertainment from incomplete vaccination provider identification and unknown medical record completeness) and do not address potential differential noncoverage or nonresponse bias over time (10). Second, weighted linear regression analyses using national data did not account for methodologic changes in sampling frames. Although vaccination estimates from landline only (2006–2010) and dual sampling frames (2011–2012) might not be comparable, prior methodologic assessment suggests that the addition of cellular telephone numbers beginning in 2011 should have had limited effects on annual national coverage estimates. Finally, estimates for particular states and reporting areas and for racial/ethnic populations with sample sizes <1,000 might be unreliable. For HPV coverage analyses by state and sex, small sample sizes decrease the power to detect differences.

Achieving high vaccination coverage among adolescents is feasible, and progress is evident for most vaccines. Lack of progress with HPV vaccination among females warrants immediate action by health-care providers, parents, public health agencies, and other immunization stakeholders. Through the VFC program, eligible children and teens can receive recommended vaccines at no cost to their families for the vaccines. Additional efforts are needed to ensure that health-care providers administer recommended HPV and meningococcal vaccinations to boys and girls during the same visits when Tdap is given. Providers, parents, and adolescents should use every health-care visit, whether for health problems, well-checks, or physicals for sports, school, or camp, as an opportunity to review adolescents’ immunization histories and ensure that every adolescent is fully vaccinated on time with every recommended vaccine (1,4,6).

## Figures and Tables

**FIGURE:**
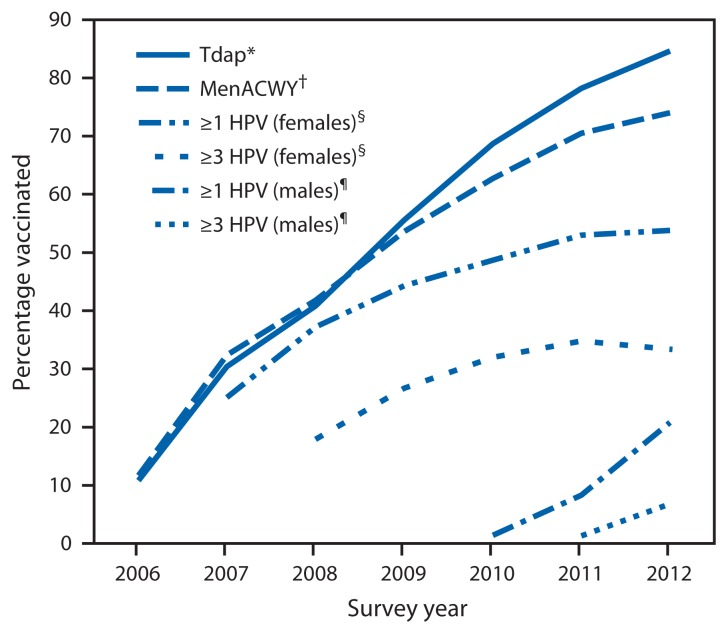
Estimated vaccination coverage with selected vaccines and doses among adolescents aged 13–17 years, by survey year — National Immunization Survey–Teen, United States, 2006–2012 **Abbreviations:** Tdap = tetanus toxoid, reduced diphtheria toxoid, and acellular pertussis; MenACWY = meningococcal conjugate; HPV = human papillomavirus; ACIP = Advisory Committee on Immunization Practices. * ≥1 dose Tdap vaccine on or after age 10 years. ^†^ ≥1 dose MenACWY vaccine. ^§^ HPV vaccine, either bivalent or quadrivalent, among females. ACIP recommends either bivalent or quadrivalent vaccine for females. ^¶^ HPV vaccine, either bivalent or quadrivalent, among males. ACIP recommends the quadrivalent vaccine for males; however, some males might have received bivalent vaccine.

**TABLE 1:** Estimated vaccination coverage with selected vaccines among adolescents aged 13–17* years, by age when interviewed — National Immunization Survey–Teen (NIS-Teen), United States, 2011–2012

	Age when interviewed (yrs) — 2012										Total			
		
	13 (n = 3,937)		14 (n = 3,961)		15 (n = 3,892)		16 (n = 3,825)		17 (n = 3,584)		2012 (N = 19,199)		2011 (N = 23,564)	
							
Vaccine	%	(95% CI)†	%	(95% CI)	%	(95% CI)	%	(95% CI)	%	(95% CI)	%	(95% CI)	%	(95% CI)
**Tdap**§ **≥1 dose**	85.3	(±2.1)	85.7	(±2.1)	84.9	(±2.0)	83.8	(±2.1)	83.3	(±2.0)	**84.6**	**(±0.9)** ¶	**78.2**	**(±0.9)**
MenACWY** **≥1 dose**	72.5	(±2.6)	73.4	(±2.6)	75.3	(±2.4)	74.6	(±2.7)	74.2	(±2.7)	**74.0**	**(±1.1)** ¶	**70.5**	**(±1.0)**
**HPV**†† **vaccine coverage**														
Females														
≥1 dose	46.8	(±4.0)	49.4	(±4.2)	53.9	(±3.9)§§	55.8	(±4.4)§§	64.2	(±4.3)§§	**53.8**	**(±1.9)**	**53.0**	**(±1.7)**
≥2 doses	31.5	(±3.5)	36.8	(±4.0)	45.3	(±3.8)§§	47.4	(±4.3)§§	56.7	(±4.6)§§	**43.4**	**(±1.9)**	**43.9**	**(±1.7)**
≥3 doses	20.2	(±3.0)	28.7	(±3.8)§§	35.3	(±3.6)§§	39.1	(±4.0)§§	44.5	(±4.7)§§	**33.4**	**(±1.7)**	**34.8**	**(±1.6)**
Males														
≥1 dose	19.5	(±3.1)	22.2	(±3.6)	20.9	(±3.3)	21.2	(±3.4)	20.3	(±3.6)	**20.8**	**(±1.5)** ¶	**8.3**	**(±1.0)**
≥2 doses	12.4	(±2.7)	13.0	(±2.8)	13.2	(±2.9)	12.9	(±2.9)	12.0	(±2.8)	**12.7**	**(±1.3)** ¶	**3.8**	**(±0.7)**
≥3 doses	6.6	(±1.8)	5.9	(±2.1)	8.1	(±2.5)	6.0	(±1.6)	7.3	(±2.5)	**6.8**	**(±1.0)** ¶	**1.3**	**(±0.3)**
**HPV**†† **3-dose series completion**¶¶														
Females	49.9	(±6.4)	64.4	(±6.9)§§	68.9	(±5.2)§§	73.1	(±4.7)§§	72.4	(±6.0)§§	**66.7**	**(±2.6)** ¶	**70.7**	**(±2.3)**
Males	47.9	(±11.0)	40.2	(±11.6)	48.3	(±10.3)	38.5	(±9.8)	50.3	(±11.8)	**45.1**	**(±5.0)** ¶	**28.1**	**(±6.5)**
MMR*** **≥2 doses**	91.2	(±1.8)	91.9	(±1.9)	92.0	(±1.5)	90.7	(±1.7)	91.1	(±1.5)	**91.4**	**(±0.8)**	**91.1**	**(±0.7)**
**Hepatitis B ≥3 doses**	93.0	(±1.6)	93.6	(±1.8)	93.4	(±1.4)	91.6	(±1.6)	92.6	(±1.4)	**92.8**	**(±0.7)**	**92.3**	**(±0.7)**
**Varicella**														
History of varicella disease†††	20.5	(±2.4)	22.0	(±2.2)	31.1	(±2.6)§§	34.9	(±2.7)§§	45.1	(±3.1)§§	**30.6**	**(±1.2)** ¶	**36.6**	**(±1.1)**
Among adolescents with no history of disease														
≥1 dose	97.2	(±1.0)	95.0	(±2.1)	95.3	(±1.5)§§	93.3	(±1.7)§§	91.3	(±2.1)§§	**94.7**	**(±0.8)** ¶	**92.3**	**(±1.0)**
≥2 doses	78.9	(±2.6)	75.6	(±3.1)	75.8	(±3.0)	71.9	(±3.4)§§	70.6	(±3.7)§§	**74.9**	**(±1.4)** ¶	**68.3**	**(±1.4)**
History of disease or received ≥2 doses varicella vaccine	83.2	(±2.1)	80.9	(±2.5)	83.3	(±2.2)	81.7	(±2.3)	83.9	(±2.1)	**82.6**	**(±1.0)** ¶	**79.9**	**(±1.0)**

**Abbreviations:** CI = confidence interval; Tdap = tetanus toxoid, reduced diphtheria toxoid, and acellular pertussis; MenACWY = meningococcal conjugate; HPV = human papillomavirus; MMR = measles, mumps, and rubella.

* Adolescents (N = 19,199) in the 2012 NIS-Teen were born during January 6, 1994–February 18, 2000.

† Estimates with 95% CI widths >20 might not be reliable.

§ Includes percentages receiving Tdap vaccine on or after age 10 years.

¶ Statistically significant difference (p<0.05) compared with 2011 NIS-Teen overall estimates.

** Includes percentages receiving MenACWY or meningococcal–unknown type vaccine.

†† HPV vaccine, either quadrivalent or bivalent. Percentage reported among females (n = 9,058) and males (n =10,141). Some adolescents might have received more than the recommended 3 doses of HPV vaccine.

§§ Statistically significant difference (p<0.05) in estimated vaccination coverage by age; reference group was adolescents aged 13 years.

¶¶ The completion rate for the 3-dose HPV vaccination series represents the percentage of adolescents who received 3 doses among those who had ≥1 HPV vaccine dose and ≥24 weeks between the first dose and the interview date. The calculation was limited to 4,548 females and 1,414 males who met the criteria of having received ≥1 HPV vaccine dose and having ≥24 weeks between the first dose and the interview date.

*** ≥2 doses of MMR vaccine.

††† By parent/guardian report or provider records.

**TABLE 2:** Estimated vaccination coverage among adolescents aged 13–17 years,* by race/ethnicity,† poverty level,§ and selected vaccines and doses — National Immunization Survey–Teen (NIS-Teen), United States, 2012

	Race/Ethnicity												Poverty status			
		
	White (n = 12,930)		Black (n = 1,928)		Hispanic (n = 2,552)		American Indian/Alaska Native (n = 261)		Asian (n = 622)		Multiracial (n = 840)		Below poverty level (n = 3,136)		At or above poverty level (n = 15,466)	
								
Vaccine	%	(95% CI)¶	%	(95% CI)	%	(95% CI)	%	(95% CI)	%	(95% CI)	%	(95% CI)	%	(95% CI)	%	(95% CI)
Tdap** **≥1 dose**	84.4	(±1.0)	83.7	(±2.5)	85.4	(±2.5)	89.5	(±5.6)	84.9	(±5.4)	85.5	(±4.7)	83.6	(±2.1)	85.1	(±1.0)
**MenACWY**†† **≥1 dose**	71.3	(±1.3)	75.8	(±3.2)§§	77.6	(±3.2)§§	82.0	(±7.9)§§	79.4	(±6.4)§§	77.9	(±4.7)§§	73.2	(±2.7)	74.1	(±1.3)
**HPV**¶¶ **coverage by dose**																
Females																
≥1 dose	51.1	(±2.1)	50.1	(±5.4)	62.9	(±4.9)§§	67.7	(±15.5)§§	55.9	(±10.9)	49.9	(±9.0)	64.9	(±4.2)§§	50.4	(±2.0)
≥2 doses	41.8	(±2.1)	39.5	(±5.1)	49.3	(±5.1)§§	43.2	(±17.7)	48.1	(±11.1)	41.3	(±8.7)	51.5	(±4.4)§§	40.7	(±2.0)
≥3 doses	33.7	(±2.0)	29.0	(±4.7)	35.5	(±4.8)	36.8	(±16.5)	33.8	(±10.2)	32.1	(±8.1)	36.2	(±4.2)	32.5	(±1.9)
Males																
≥1 dose	15.2	(±1.4)	25.9	(±4.6)§§	31.7	(±4.7)§§	24.9	(±12.0)	22.3	(±8.7)	20.7	(±6.4)	29.9	(±3.9)§§	17.3	(±1.5)
≥2 doses	9.0	(±1.1)	15.6	(±3.8)§§	20.1	(±4.1)§§	NA	NA	17.1	(±7.8)§§	10.3	(±4.0)	18.8	(±3.4)§§	10.2	(±1.2)
≥3 doses	4.6	(±0.8)	5.4	(±1.9)	12.9	(±3.5)§§	NA	NA	NA	NA	5.4	(±3.0)	10.7	(±2.9)§§	5.5	(±0.9)
**HPV**¶¶ 3-dose series completion***																
Females	71.8	(±2.7)	63.7	(±7.1)§§	59.3	(±6.8)§§	55.4	(±27.4)	61.8	(±15.8)	67.8	(±11.3)	59.3	(±5.8)§§	69.9	(±2.7)
Males	45.2	(±6.2)	27.8	(±9.2)§§	52.1	(±10.3)	NA	NA	62.7	(±23.6)	38.2	(±19.2)	43.6	(±9.1)	47.2	(±5.6)
**MMR**†††≥**2 doses**	92.4	(±0.8)	91.4	(±2.3)	89.1	(±2.2)§§	95.9	(±4.2)	90.4	(±4.6)	90.4	(±3.7)	89.7	(±1.9)§§	92.0	(±0.8)
**Hepatitis B ≥3 doses**	93.7	(±0.7)	92.5	(±2.1)	91.1	(±2.1)§§	94.1	(±5.8)	92.0	(±3.8)	92.0	(±3.3)	91.3	(±1.7)§§	93.3	(±0.8)
**Varicella**																
History of varicella disease§§§	32.4	(±1.3)	27.2	(±3.3)§§	29.1	(±3.2)	38.0	(±10.8)	25.9	(±7.1)	28.8	(±5.2)	30.7	(±2.7)	30.5	(±1.3)
Among adolescents with no history of disease																
≥1 dose	95.3	(±0.8)	93.3	(±2.5)	94.1	(±2.1)	95.2	(±6.9)	93.5	(±4.4)	95.5	(±2.9)	92.5	(±2.0)§§	95.3	(±0.8)
≥2 doses	74.0	(±1.7)	75.2	(±3.9)	76.3	(±3.5)	78.4	(±11.8)	79.4	(±8.0)	75.1	(±6.3)	72.0	(±3.3)§§	75.8	(±1.5)
History of disease or received ≥2 doses varicella vaccine	82.4	(±1.2)	81.9	(±3.0)	83.2	(±2.6)	86.6	(±7.2)	84.7	(±6.2)	82.3	(±4.6)	80.6	(±2.4)	83.2	(±1.1)

**Abbreviations:** CI = confidence interval; Tdap = tetanus toxoid, reduced diphtheria toxoid, and acellular pertussis; MenACWY = meningococcal conjugate; HPV = human papillomavirus; NA = not available (estimate not reported because unweighted sample size for the denominator was <30 or 95% CI half width/estimate >0.6); MMR = measles, mumps, and rubella.

* Adolescents (N = 19,199) in the 2012 NIS-Teen were born during January 6, 1994–February 18, 2000.

† Adolescent’s race/ethnicity was reported by their parent or guardian. Adolescents identified in this report as white, black, Asian, American Indian/Alaska Native or multiracial were reported by the parent or guardian as non-Hispanic. Adolescents identified as multiracial had more than one race category selected. Adolescents identified as Hispanic might be of any race. Native Hawaiian or other Pacific Islanders were not included in the table because of small sample sizes.

§ Adolescents were classified as below poverty level if their total family income was less than the federal poverty level specified for the applicable family size and number of children aged <18 years. All others were classified as at or above the poverty level. Additional information available at http://www.census.gov/hhes/www/poverty.html. Poverty status was unknown for 597 adolescents.

¶ Estimates with 95% CI widths >20 might not be reliable.

** Includes percentages receiving Tdap vaccine on or after age 10 years.

†† Includes percentages receiving MenACWY and meningococcal–unknown type vaccine.

§§ Statistically significant difference (p<0.05) in estimated vaccination coverage by race/ethnicity or poverty level; referent groups were white, non-Hispanic adolescents and adolescents living at or above poverty level, respectively.

¶¶ HPV vaccine, either quadrivalent or bivalent. Percentage reported among females (n = 9,058) and males (n = 10,141). Some adolescents might have received more than the 3 recommended HPV vaccine doses.

*** The completion rate for the 3-dose HPV vaccination series represents the percentage of adolescents who received 3 doses among those who had ≥1 HPV vaccine dose and ≥24 weeks between the first dose and the interview date. The calculation was limited to 4,548 females and 1,414 males who met the criteria of having received ≥1 HPV vaccine dose and having ≥24 weeks between the first dose and the interview date.

††† Includes ≥2 doses of MMR vaccine.

§§§ By parent/guardian report or provider records.

**TABLE 3:** Estimated vaccination coverage with selected vaccines and doses* among adolescents aged 13–17 years,† by state/area — National Immunization Survey–Teen (NIS-Teen), United States, 2012

							Females (N = 9,058)						Males (N = 10,141)					
					
	≥2 VAR§		≥1 Tdap¶		≥1 MenACWY**		≥1 HPV††		≥2 HPV§§		≥3 HPV¶¶		≥1 HPV††		≥2 HPV§§		≥3 HPV¶¶	
									
State/Area	%	(95% CI)***	%	(95% CI)	%	(95% CI)	%	(95% CI)	%	(95% CI)	%	(95% CI)	%	(95% CI)	%	(95% CI)	%	(95% CI)
**United States overall**	**74.9**	**(±1.4)** †††	**84.6**	**(±0.9)** †††	**74.0**	**(±1.1)** †††	**53.8**	**(±1.9)**	**43.4**	**(±1.9)**	**33.4**	**(±1.7)**	**20.8**	**(±1.5)** †††	**12.7**	**(±1.3)** †††	**6.8**	**(±1.0)** †††
**Northeast**	82.0	(±2.5)†††	90.5	(±1.5)†††	85.3	(±1.8)†††	58.2	(±3.7)	51.4	(±3.7)	40.4	(±3.7)	21.2	(±2.8)†††	12.8	(±2.3)†††	6.4	(±1.7)†††
Connecticut	93.5	(±4.3)	89.3	(±4.8)	88.8	(±3.7)†††	57.6	(±10.3)	53.9	(±10.4)	43.6	(±10.5)	20.3	(±6.7)	14.6	(±6.0)	8.5	(±4.6)
Maine	75.6	(±7.4)	79.5	(±5.9)†††	73.7	(±6.1)†††	61.7	(±9.4)	53.4	(±9.7)	41.8	(±9.6)	25.3	(±7.9)	17.4	(±7.0)	12.1	(±6.2)
Massachusetts	88.8	(±4.7)	95.7	(±2.4)	89.2	(±3.7)	69.3	(±7.9)	58.9	(±8.9)	43.0	(±9.1)	25.5	(±7.9)	10.4	(±5.0)	NA	NA
New Hampshire	92.9	(±3.9)	96.3	(±2.2)	83.1	(±5.6)	52.2	(±10.6)§§§	43.6	(±10.4)	34.5	(±9.7)	20.5	(±7.3)	12.2	(±5.5)	NA	NA
New Jersey	73.8	(±7.2)	90.9	(±4.0)†††	91.6	(±3.9)	54.6	(±9.7)	44.9	(±9.7)	31.6	(±8.5)	19.8	(±7.9)	10.7	(±5.6)	NA	NA
New York	74.4	(±5.1)	90.3	(±2.9)	78.5	(±4.1)	56.0	(±7.1)†††	50.5	(±7.2)	39.7	(±7.2)	17.9	(±5.1)†††	12.3	(±4.6)	NA	NA
City of New York	70.2	(±7.5)	86.4	(±4.5)	75.3	(±5.8)	53.6	(±8.9)	49.0	(±9.0)	37.3	(±8.9)	27.3	(±9.5)†††	19.2	(±8.7)	NA	NA
Rest of state	77.1	(±6.9)	92.7	(±3.8)	80.5	(±5.5)†††	57.5	(±10.2)†††	51.4	(±10.3)†††	41.3	(±10.3)	12.1	(±5.5)	NA	NA	NA	NA
Pennsylvania	90.4	(±4.3)	88.4	(±3.4)†††	89.4	(±3.6)†††	57.4	(±8.0)	52.1	(±8.2)	44.6	(±8.2)	21.9	(±6.0)†††	13.2	(±4.8)	5.3	(±2.8)
Philadelphia County	90.0	(±4.7)†††	87.2	(±4.7)	92.9	(±3.7)	76.2	(±8.2)	68.5	(±9.3)	51.9	(±10.3)	46.7	(±9.8)†††	27.5	(±9.0)†††	NA	NA
Rest of state	90.4	(±4.8)	88.6	(±3.8)†††	88.9	(±4.0)	55.0	(±9.0)	50.0	(±9.2)	43.6	(±9.2)	18.8	(±6.6)†††	11.4	(±5.3)	NA	NA
Rhode Island	93.3	(±3.8)†††	94.0	(±2.9)†††	94.3	(±2.9)†††	73.7	(±9.4)	67.8	(±9.8)	57.7	(±10.0)	55.2	(±9.2)†††	34.8	(±8.7)†††	17.7	(±6.3)
Vermont	92.4	(±3.8)†††	93.1	(±3.6)	72.6	(±6.1)	66.4	(±9.0)	58.0	(±9.3)	46.2	(±9.6)	25.7	(±8.2)	19.4	(±7.8)	10.6	(±5.6)
**Midwest**	72.9	(±2.7)	82.9	(±2.0)†††	71.9	(±2.2)	50.5	(±3.5)	39.4	(±3.4)	31.1	(±3.2)	18.1	(±2.7)†††	10.7	(±2.1)†††	5.4	(±1.7)†††
Illinois	63.4	(±7.2)	77.3	(±5.4)	67.7	(±6.0)	41.2	(±8.5)	28.5	(±7.7)§§§	21.1	(±6.3)§§§	24.3	(±7.8)†††	15.0	(±6.8)†††	NA	NA
City of Chicago	72.2	(±8.2)	78.5	(±6.1)†††	77.0	(±6.2)	61.4	(±10.4)†††	44.5	(±11.0)	37.8	(±10.8)	40.2	(±10.5)	27.8	(±10.1)	17.0	(±9.3)
Rest of state	60.9	(±8.9)	77.0	(±6.5)	65.4	(±7.2)	36.2	(±10.1)§§§	24.5	(±9.1)§§§	16.9	(±7.3)§§§	20.5	(±9.4)	NA	NA	NA	NA
Indiana	84.8	(±6.7)	94.4	(±3.0)	92.0	(±3.8)	48.4	(±9.9)	42.7	(±9.7)	35.2	(±9.1)	17.2	(±7.4)	10.8	(±5.9)	NA	NA
Iowa	62.1	(±8.5)	77.8	(±5.9)	64.4	(±6.7)	57.5	(±9.6)	46.4	(±9.8)	35.6	(±9.3)	19.4	(±7.8)	13.5	(±6.4)	NA	NA
Kansas	78.7	(±7.0)†††	92.2	(±3.3)†††	55.9	(±7.3)	42.7	(±10.5)	32.8	(±9.8)	25.1	(±9.3)	13.5	(±6.9)	11.1	(±6.5)	NA	NA
Michigan	87.4	(±5.0)	84.2	(±4.8)†††	87.5	(±4.2)†††	48.1	(±9.7)	39.2	(±9.6)	32.2	(±9.3)	13.1	(±6.9)	NA	NA	NA	NA
Minnesota	82.9	(±6.6)	85.6	(±6.1)	66.6	(±6.8)	59.4	(±10.3)	46.0	(±10.7)	33.1	(±9.9)	15.2	(±7.6)	NA	NA	NA	NA
Missouri	53.6	(±9.7)	88.0	(±4.8)†††	58.3	(±7.6)	51.6	(±10.5)	40.4	(±10.1)	34.5	(±9.7)	21.7	(±9.9)	NA	NA	NA	NA
Nebraska	82.2	(±6.4)	81.4	(±5.8)	75.5	(±6.1)	67.5	(±10.0)	58.3	(±10.7)†††	37.3	(±10.0)	19.6	(±6.9)	11.6	(±5.0)	7.0	(±3.7)
North Dakota	68.6	(±8.9)	89.5	(±5.0)	88.1	(±4.9)	60.3	(±9.8)	49.7	(±10.0)	40.9	(±9.6)	18.6	(±7.4)	13.1	(±6.8)	NA	NA
Ohio	62.0	(±8.8)	73.8	(±6.7)	66.4	(±6.9)	56.4	(±10.4)	39.5	(±10.7)	31.9	(±10.5)	15.2	(±6.7)	6.9	(±3.9)	NA	NA
South Dakota	43.7	(±9.0)	65.9	(±6.5)	40.0	(±6.8)	51.0	(±10.1)	46.5	(±10.1)	31.8	(±9.3)§§§	19.8	(±8.2)	10.7	(±6.1)	NA	NA
Wisconsin	87.9	(±5.4)	89.8	(±4.4)	74.4	(±6.2)	50.5	(±10.8)§§§	45.0	(±10.7)§§§	37.5	(±10.5)	19.3	(±8.0)	10.3	(±5.8)	NA	NA
**South**	73.3	(±2.1)†††	81.2	(±1.5)†††	71.0	(±1.8)†††	48.9	(±2.9)	39.5	(±2.7)	29.9	(±2.5)	20.1	(±2.3)†††	12.0	(±1.9)†††	6.2	(±1.2)†††
Alabama	68.1	(±8.5)†††	81.7	(±6.0)	60.5	(±7.1)	46.6	(±10.4)	36.9	(±10.1)	31.1	(±9.9)	17.8	(±9.3)	NA	NA	NA	NA
Arkansas	53.3	(±8.4)	64.4	(±6.8)†††	37.5	(±7.0)†††	41.2	(±10.7)	32.4	(±10.0)	18.3	(±7.2)	12.7	(±6.6)	NA	NA	NA	NA
Delaware	84.9	(±6.3)	77.8	(±5.9)	78.0	(±6.2)	67.2	(±9.8)	64.5	(±9.9)	50.4	(±10.2)	26.2	(±7.5)†††	17.9	(±6.7)†††	10.7	(±4.9)
District of Columbia	92.3	(±5.0)	84.5	(±5.2)	92.1	(±3.3)	57.8	(±10.1)	52.8	(±10.1)	38.5	(±9.7)	33.8	(±9.7)	12.3	(±6.1)	4.8	(±2.5)
Florida	73.3	(±8.5)	86.8	(±5.1)†††	68.6	(±6.8)	39.4	(±10.1)	33.4	(±9.6)	25.3	(±8.8)	21.4	(±9.3)	15.4	(±8.2)	NA	NA
Georgia	89.3	(±5.2)	80.5	(±6.0)†††	73.1	(±6.8)	52.3	(±10.8)	36.8	(±9.8)	29.0	(±9.0)	19.5	(±8.5)†††	8.7	(±4.7)	NA	NA
Kentucky	57.3	(±8.4)†††	80.0	(±5.6)†††	62.9	(±6.8)	51.2	(±10.6)	43.5	(±10.5)	34.9	(±9.9)	NA	NA	NA	NA	NA	NA
Louisiana	84.8	(±5.2)	89.8	(±3.7)	90.8	(±3.6)	62.1	(±8.6)	52.6	(±9.1)	40.5	(±9.0)	20.6	(±8.2)	12.6	(±6.9)	NA	NA
Maryland	80.4	(±6.9)†††	78.1	(±6.6)	74.9	(±6.9)	42.7	(±10.9)	39.3	(±10.5)	30.9	(±9.4)	20.2	(±7.5)	13.8	(±6.4)	NA	NA
Mississippi	48.1	(±9.7)†††	53.5	(±7.3)†††	40.7	(±7.1)	39.7	(±10.6)	22.3	(±7.7)	12.1	(±5.9)	20.9	(±9.2)	11.2	(±6.4)	NA	NA
North Carolina	66.7	(±7.9)	87.9	(±4.5)†††	68.2	(±6.4)	53.3	(±9.7)	46.5	(±9.8)	35.5	(±9.5)	18.8	(±7.1)	11.8	(±5.7)	8.6	(±5.0)
Oklahoma	65.1	(±7.7)†††	77.1	(±5.6)†††	63.8	(±6.7)	55.1	(±9.5)	49.5	(±9.6)	38.4	(±9.4)	24.4	(±7.6)†††	14.8	(±6.0)	10.6	(±5.4)
South Carolina	58.3	(±8.6)	64.9	(±7.2)	58.5	(±7.3)	41.9	(±10.6)	31.6	(±9.8)	26.6	(±9.5)	18.1	(±8.8)	15.9	(±8.5)	NA	NA
Tennessee	70.8	(±8.7)	77.4	(±6.2)†††	69.4	(±6.7)	54.3	(±11.0)	40.9	(±10.7)	28.6	(±9.4)	20.3	(±8.8)	NA	NA	NA	NA
Texas	79.1	(±3.9)	82.5	(±3.3)	84.6	(±3.3)†††	51.2	(±5.8)	41.2	(±5.7)	30.3	(±5.3)	24.0	(±5.0)†††	14.2	(±4.1)†††	7.0	(±2.4)†††
Bexar County	72.5	(±8.5)	78.6	(±7.2)	83.6	(±6.0)	43.0	(±10.4)	33.4	(±9.8)	26.3	(±9.3)	16.6	(±8.3)	NA	NA	NA	NA
City of Houston	77.6	(±7.3)	82.5	(±5.7)	87.6	(±4.5)	55.8	(±9.4)	46.0	(±9.6)	36.8	(±9.5)	38.0	(±10.1)†††	23.8	(±9.0)	15.1	(±7.9)
Rest of state	79.8	(±4.5)	82.9	(±3.7)	84.4	(±3.8)	51.5	(±6.7)	41.4	(±6.6)	30.1	(±6.1)	23.3	(±5.7)†††	13.8	(±4.7)	6.5	(±2.7)
Virginia	69.1	(±7.7)†††	88.7	(±4.3)†††	62.1	(±7.4)	50.9	(±10.9)	38.0	(±10.3)	27.9	(±9.2)	12.1	(±5.8)	NA	NA	NA	NA
West Virginia	61.5	(±9.3)†††	68.2	(±7.1)	64.1	(±7.4)	45.2	(±10.6)	41.2	(±10.6)	36.1	(±10.2)	18.3	(±8.5)†††	NA	NA	NA	NA
**West**	73.8	(±3.6)†††	87.4	(±2.2)†††	72.5	(±3.1)	61.4	(±4.7)	47.2	(±4.9)	36.2	(±4.7)	24.3	(±4.1)†††	15.6	(±3.6)†††	9.4	(±2.9)
Alaska	73.6	(±7.5)†††	77.1	(±5.0)†††	52.7	(±6.2)	56.1	(±9.3)	46.3	(±9.6)	31.4	(±8.8)	14.1	(±5.6)	7.5	(±3.7)	NA	NA
Arizona	73.8	(±6.8)†††	87.5	(±4.5)	85.5	(±5.0)	54.3	(±9.5)	43.4	(±9.5)	36.9	(±9.3)	19.7	(±7.0)†††	12.8	(±5.8)	NA	NA
California	75.3	(±6.2)†††	89.4	(±3.8)†††	76.0	(±5.5)	65.0	(±8.3)	48.4	(±8.8)	35.8	(±8.4)	29.4	(±7.4)†††	19.3	(±6.4)	11.7	(±5.2)
Colorado	81.6	(±6.6)	93.2	(±3.5)†††	73.2	(±6.6)	61.4	(±10.8)†††	44.9	(±11.3)	38.0	(±11.2)	31.3	(±12.6)†††	NA	NA	NA	NA
Hawaii	76.0	(±6.6)	74.1	(±5.9)	70.4	(±6.3)	64.6	(±9.4)	58.1	(±9.8)	43.4	(±9.7)	43.1	(±9.7)†††	27.5	(±8.8)	15.6	(±7.6)
Idaho	57.0	(±8.7)	64.5	(±6.1)	63.2	(±6.3)†††	51.3	(±9.5)	41.6	(±9.6)	27.8	(±8.2)	16.2	(±7.5)	NA	NA	NA	NA
Montana	61.3	(±8.9)	90.2	(±3.8)	58.6	(±6.6)†††	55.1	(±9.8)	46.5	(±10.0)	41.6	(±10.1)	16.8	(±7.0)	10.0	(±5.9)	NA	NA
Nevada	69.4	(±7.8)†††	86.3	(±5.0)	66.3	(±6.3)	62.5	(±9.5)	44.6	(±10.2)	37.2	(±10.2)	11.6	(±5.5)	NA	NA	NA	NA
New Mexico	60.5	(±8.2)	82.6	(±5.6)	54.2	(±7.0)§§§	51.1	(±10.1)	38.7	(±9.4)	30.3	(±8.7)	20.2	(±8.1)	12.8	(±7.0)	NA	NA
Oregon	75.6	(±6.2)†††	86.0	(±4.5)	58.3	(±6.3)	58.5	(±9.3)	46.7	(±9.5)	38.6	(±9.3)	14.5	(±5.9)	7.2	(±4.2)	NA	NA
Utah	59.2	(±8.7)	81.5	(±6.3)	56.5	(±7.0)	44.3	(±10.4)	39.0	(±10.0)	24.1	(±8.4)	NA	NA	NA	NA	NA	NA
Washington	73.9	(±8.4)	86.0	(±5.1)†††	71.2	(±6.6)	64.5	(±10.1)	54.6	(±10.1)	43.5	(±9.8)	14.9	(±6.2)	9.6	(±5.4)	NA	NA
Wyoming	88.8	(±5.6)	85.4	(±4.8)	59.0	(±6.6)	53.9	(±10.0)	41.4	(±9.6)	30.3	(±8.7)	11.2	(±4.9)	NA	NA	NA	NA
**Territory**																		
U.S. Virgin Islands	75.6	(±4.4)†††	72.0	(±4.5)†††	38.1	(±4.8)	28.7	(±6.5)	16.4	(±5.5)	9.1	(±4.4)	10.5	(±4.5)	NA	NA	NA	NA

**Abbreviations:** CI = confidence interval; VAR = varicella; Tdap = tetanus toxoid, reduced diphtheria toxoid, and acellular pertussis; MenACWY = meningococcal conjugate; HPV = human papillomavirus; NA = not available (estimate not reported because unweighted sample size for the denominator was <30 or 95% CI half width/estimate >0.6).

* Vaccination estimates for additional measures, including ≥2 doses MMR, ≥3 doses hepatitis B, and ≥1 dose varicella vaccines are available at http://www.cdc.gov/vaccines/stats-surv/nis/default.htm#nisteen.

† Adolescents (N = 19,199) in the 2012 NIS-Teen were born during January 6, 1994–February 18, 2000.

§ ≥2 doses of VAR vaccine among adolescents without a reported history of varicella disease.

¶ ≥1 dose Tdap vaccine on or after age 10 years.

** ≥1 dose of MenACWY or meningococcal–unknown type vaccine.

†† ≥1 dose of HPV vaccine, either quadrivalent or bivalent. For ≥1, ≥2, and ≥3 dose measures, separate percentages are reported among females only (N = 9,058) and among males only (N = 10,141).

§§ ≥2 doses of HPV vaccine, either quadrivalent or bivalent.

¶¶ ≥3 doses of HPV vaccine, either quadrivalent or bivalent. Some adolescents might have received more than the recommended 3 HPV vaccine doses.

*** Estimates with 95% CI half-widths >10 might not be reliable.

††† Statistically significant (p<0.05) percentage point increase from 2011.

§§§ Statistically significant (p<0.05) percentage point decrease from 2011.

## Footnotes

* Adolescents who receive their first MenACWY vaccine dose as routinely recommended at age 11–12 years should receive a booster dose at 16 years. Adolescents who receive their first dose at ages 13–15 years should receive a booster dose at ages 16–18 years, with a minimum interval of ≥8 weeks between doses. Adolescents who receive a MenACWY vaccine dose at age ≥16 years do not need a booster dose.

† Eligible participants were born during January 1994–February 2000. Except as noted, coverage for ≥1 and ≥2 varicella vaccine doses were obtained among persons with no history of varicella disease. HPV vaccination coverage represents receipt of any HPV vaccine and does not distinguish between bivalent or quadrivalent vaccines. Some adolescents, both males and females, might have received more than the 3 recommended HPV vaccine doses. Influenza vaccination coverage data are not included in this report.

§ Includes Tdap vaccines received on or after age 10 years.

¶ Six areas that received federal Section 317 immunization funds were sampled separately: District of Columbia; Chicago, Illinois; New York, New York; Philadelphia County, Pennsylvania; Bexar County, Texas; and Houston, Texas.

** Sampling was conducted based on landline telephone sampling frame only and included St. Croix, St. Thomas, St. John, and Water Island.

†† All identified cellular-telephone households were eligible for interview. Sampling weights were adjusted from dual-frame (landline and cellular telephone), nonresponse, noncoverage, and overlapping samples of mixed telephone users. A description of NIS-Teen dual-frame survey methodology and its effect on reported vaccination estimates is available at http://www.cdc.gov/vaccines/stats-surv/nis/dual-frame-sampling-08282012.htm.

§§ In 2012, the Council of American Survey Research Organizations (CASRO) landline response rate was 55.1%. A total of 14,133 adolescents with vaccination provider-reported vaccination records were included, representing 62% of all adolescents from the landline sample with completed household interviews. The cellular-telephone sample CASRO response rate was 23.6%. A total of 5,066 adolescents with vaccination provider-reported vaccination records are included, representing 56.4% of all adolescents from the cellular-telephone sample with completed household interviews. The CASRO response rate is the product of three other rates: 1) the resolution rate (the proportion of telephone numbers that can be identified as either for a business or residence), 2) the screening rate (the proportion of qualified households that complete the screening process), and 3) the cooperation rate (the proportion of contacted eligible households for which a completed interview is obtained).

¶¶ Adolescents from the U.S. Virgin Islands (262 females and 285 males) were excluded from the national estimates.

*** Information available at ftp://ftp.cdc.gov/pub/health_statistics/nchs/dataset_documentation/nis/nisteenpuf11_dug.pdf.

††† The completion rate for the 3-dose HPV vaccination series represents the percentage of adolescents who received 3 doses among those who had ≥1 HPV vaccine dose and ≥24 weeks between the first dose and the interview date. The calculation was limited to 4,548 females and 1,414 males who met the criteria of having received ≥1 HPV vaccine dose and having ≥24 weeks between the first dose and the interview date.

§§§ Adolescents were classified as below federal poverty level if their total family income was less than the federal poverty level specified for the applicable family size and number of children aged <18 years. All others were classified as at or above the poverty level. Poverty status was unknown for 597 adolescents. Additional information available at: http://www.census.gov/hhes/www/poverty.

¶¶¶ Additional information available at http://www.immunize.org/laws.

**** Information available at http://www.cdc.gov/pertussis/outbreaks/trends.html.

†††† Children aged ≤18 years who are Medicaid-eligible, uninsured, or American Indian/Alaska Native (as defined by the Indian Health Care Improvement Act) are entitled to receive vaccines from providers through the VFC program. Children categorized as “underinsured” (because their health plans do not include coverage for recommended vaccinations) may receive VFC vaccines if they are served by a rural health clinic or federally qualified health center or under an approved deputization agreement. Additional information is available at http://www.cdc.gov/vaccines/programs/vfc/index.html.

§§§§ Patient Protection and Affordable Care Act of 2010. Pub. L. No. 114-48 (March 23, 2010), as amended through May 1, 2010. Available at http://www.healthcare.gov/law/full/index.html.
